# The *Odocoileus virginianus* Femur: Mechanical Behavior and Morphology

**DOI:** 10.1371/journal.pone.0146611

**Published:** 2016-01-12

**Authors:** Mark J. Hedgeland, Morgan A. Libruk, Nicole C. Corbiere, Mario J. Ciani, Laurel Kuxhaus

**Affiliations:** 1 Mechanical & Aeronautical Engineering, Clarkson University, Potsdam, New York, United States of America; 2 Physician Assistant Studies, Clarkson University, Potsdam, New York, United States of America; 3 Occupational Therapy, Clarkson University, Potsdam, New York, United States of America; Faculty of Animal Sciences and Food Engineering, University of São Paulo, BRAZIL

## Abstract

Biomechanical research relies heavily on laboratory evaluation and testing with osseous animal structures. While many femora models are currently in use, including those of the European red deer (*Cervus elaphus*), the *Odocoileus virginianus* femur remains undocumented, despite its regional abundance in North America. The objective of this study was to compare biomechanical and morphological properties of the *Odocoileus virginianus* femur with those of the human and commonly used animal models. Sixteen pairs of fresh-frozen cervine femora (10 male, 6 female, aged 2.1 ± 0.9 years) were used for this study. Axial and torsional stiffnesses (whole bone) were calculated following compression and torsion to failure tests (at rates of 0.1 mm/sec and 0.2°/sec). Lengths, angles, femoral head diameter and position, periosteal and endosteal diaphyseal dimensions, and condylar dimensions were measured. The results show that the cervine femur is closer in length, axial and torsional stiffness, torsional strength, and overall morphology to the human femur than many other commonly used animal femora models; additional morphological measurements are comparable to many other species’ femora. The distal bicondylar width of 59.3mm suggests that cervine femora may be excellent models for use in total knee replacement simulations. Furthermore, the cervine femoral head is more ovoid than other commonly-used models for hip research, making it a more suitable model for studies of hip implants. Thus, with further, more application-specific investigations, the cervine femur could be a suitable model for biomechanical research, including the study of ballistic injuries and orthopaedic device development.

## Introduction

Biomechanical research relies on animal models for convenient and cost-effective preliminary evaluation. In particular, accurate proxies are essential to orthopaedic surgical device development, including implant prototypes [1,2], joint replacements, other devices such as suture anchors [3], and accurate representations of ballistic injury. Comparing the morphological and biomechanical characteristics of animal bones and soft tissue structures with those of humans is the basis for development of suitable models for both *in vivo* and *in situ* evaluation of devices and injury models.

Musculoskeletal structures of ovine and porcine species are most commonly used to evaluate orthopaedic devices [4]; bovine, canine, caprine, and laprine species are also used [5–7]. Although the bones of these other species have similar microscopic compositional properties as human bone, macroscopic dissimilarities limit the applicability of mechanical testing results [4]. While synthetic models (e.g., Sawbones) are available, they lack the complex compositional properties of living bone and are therefore not ideal [8, 9].

Despite the extensive use of animal femora in biomechanical research [4–7, 10–12], the caprine, ovine, canine and bovine are all shorter than human femora [4, 5, 10, 13]. Furthermore, differences in femoral angles, head offset and cross-sectional dimensions between canines and humans limits the use of the canine femur as a model for hip arthroplasty implants [5,7]. In particular, the human femoral head is ovoid in shape, and canine femoral heads, often used to evaluate hip replacements, are more spherical; this limits the generalizability of such test results. The well-studied ovine, caprine, porcine and canine models have distal femoral dimensions such as intercondylar size and condylar widths that are much smaller than the human dimensions [6, 12], making them suboptimal knee models. Thus, there is a need for an improved femur model for *in situ* evaluation of orthopaedic devices.

Recent studies have concluded that the *Cervus elaphus* femur has morphological and biomechanical properties similar to the human femur [4], and that because of this, are acceptable models for ballistics research [14]. Since *Odocoileus virginianus* (white-tailed deer) tibiae and vertebrae have demonstrated suitability as models for human tibiae and vertebrae [15, 16], and are regionally abundant in North America, the goal of this study was to quantify the biomechanical properties and morphology of the *Odocoileus virginianus* femur and compare it to the available literature values of human, *Cervus elaphus*, and other commonly-used animal femora [14]. Based on the results of previous studies of other cervine bones, we hypothesize that the cervine femur will be as suitable a proxy to the human femur as currently-used animal models.

## Methods

### Specimens and Preparation

Sixteen pairs (10 male, 6 female) of post-mortem fresh frozen *Odocoileus virginianus* femora were obtained from Nolt’s Custom Meat Cutting (Lowville, NY). Specimens obtained were byproducts of meat-processing. Given that no live animals or human subjects data were used in this study, and the specimens were purchased from a meat-processor, ethical approval by the Institutional Animal Care and Use Committee was not necessary per the NIH Public Health Service Policy on Humane Care and Use of Laboratory Animals [17]. The average estimated age of each specimen was 2.1 ± 0.9 years; age estimates are accurate to the nearest 6 months as estimated by the meat processor. The 16 left femora were used for biomechanical testing while the 16 right femora were used for morphological study. Soft tissue structures were excised with a stainless steel scalpel prior to use. All specimens were evaluated after thawing. All specimens were sprayed with a saline solution (0.159 M) and wrapped in a saline dampened cloth when not being measured or tested.

### Biomechanical Evaluation

**Fig 1:**
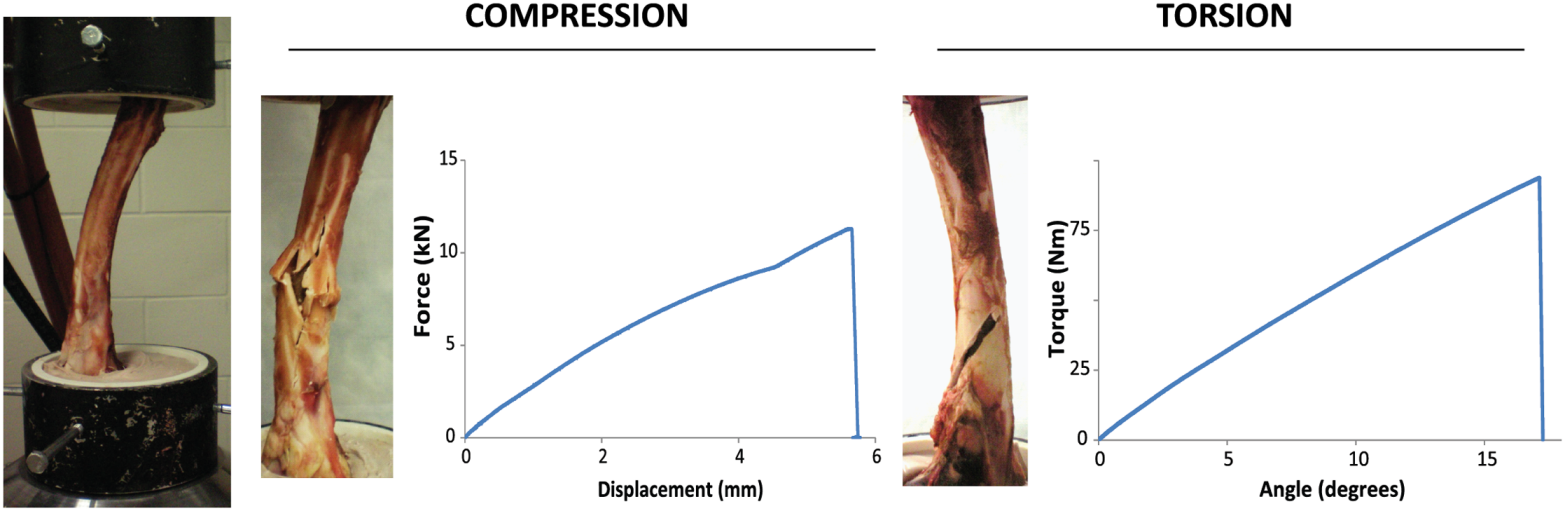
Potted specimen (left), representative axial compression (middle) and torsional (right) fractures with respective load- and torque-displacement curves.

After thawing, the 16 left femora were potted at a 10° angle in the frontal plane using Bondo^™^ (Auto Body Filler, 3M, St. Paul, MN) as a rigid support material around the diaphyses of each bone, (Fig 1) closely following the methods described in [10] and [11]. Eight of the sixteen femora were chosen for monotonic compression and the remaining eight for torsional testing. The sets of eight were chosen quasi-randomly to ensure balanced age and gender distribution. Femora used in biomechanical evaluation were frozen again after potting and thawed just prior to failure testing. Compression and torsion were performed about the biomechanical axes of the specimens to replicate *in vivo* loading conditions and enable direct comparison to previously-published results. Compression was performed at a rate of 0.1 mm/sec and torsion at 0.2°/sec to failure using a load frame (MTS 809 Axial/Torsion Test System, MTS Systems Corp., Eden Prairie, MN) and built-in load cell (Force Xncr, MTS Systems Corp., Eden Prairie, MN). In torsion, the proximal end of the femur was externally rotated relative to the fixed distal end. Failure was determined by auditory and visual observation; the load-displacement data from each trial was analyzed (Fig 1).The stiffnesses (Eqs (1) and (2)) and ultimate strength (maximum load before failure) of each specimen were calculated in MATLAB (R2012b, MathWorks, Natick, MA). Stiffness was determined as the gradient of the initial linear portion of the load-displacement curve.

$$K_{A}=\Delta F/\Delta \delta$$ (1)

where K_A_ is whole-bone axial stiffness, F is applied load, and δ is axial deformation

$$K_{T}=\Delta M/\Delta \theta$$ (2)

where K_T_ is whole-bone torsional stiffness, M is applied moment, and θ is rotation.

### Morphological Evaluation

**Fig 2:**
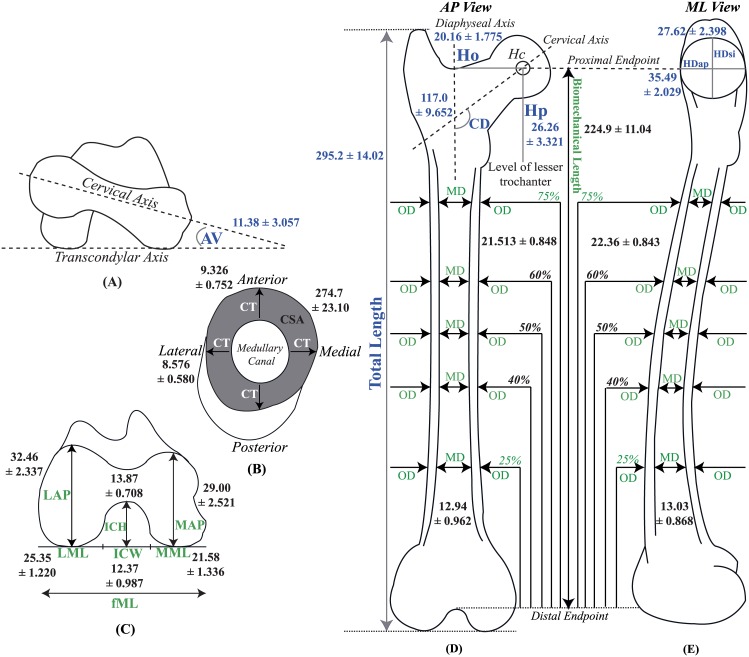
Morphologic dimensions (A) AV angle (B) Cross-sectional view of diaphysis showing CT in AP and ML planes, and CSA represented by the shaded area (C) Distal femoral dimensions (D) AP view of femur showing length, HO and HP relative to HC, CD angle and OD. Dimensions shown in green were measured by hand, and those shown in blue were measured in ImageJ.

Geometric dimensions [4, 5, 7, 12, 13, 18–20] of right cervine femora were measured either by hand or with a Java-based image processing and analysis application (ImageJ64 version http://rsb.info.nih.gov) as described below and illustrated in Fig 2. Each dimension was measured three times. Hand-made measurements, in millimeters, were performed by three different individuals using inside/outside calipers and a metric ruler with the exception of biomechanical length (L_B_), which was measured using a flexible tape measure (150 cm/60 in). Photographic dimensions were measured in ImageJ three times by the same individual (three photos per each view of the femur, described below) with a metric ruler included in the photo for scale (Fujifilm FinePix Z33WP, Fujifilm, Tokyo, Japan). The camera was held perpendicular to the benchtop at a height of approximately 8 inches for each photo. The relevant lines/points of reference (cervical, diaphyseal, and transcondylar axes and head center) were inserted using an image viewer application (Preview for Mac OS, version 5.5.3 [719.31]) prior to analysis in ImageJ. In each photo, scales were set for pixels/10 mm. Dimensions were recorded to the nearest 1° or millimeter as appropriate.

Total bone length (L_T_), defined as the distance from the tip of the greater trochanter to the most distal point of the lateral condyle (Fig 2D), was measured in ImageJ using a photo of the posterior view of the femur. Biomechanical length (L_B_), defined as the distance between the trochanteric fossa and intercondylar notch (Fig 2D and 2E), was measured with a tape measure held parallel to the bench top. Anteversion angle (AV), defined as the angle created by the intersection of cervical and transcondylar axes (Fig 2A) was captured by photographing the femur in the cervine frontal plane (analogous to the transverse plane of the human femur). The femur was placed posterior side down and the camera was held perpendicular to and at the level of the bench top, pointing proximally. Cervicodiaphysceal angle (CD), defined as the angle formed by the intersection of cervical and diaphyseal axes (Fig 2D), was measured using the posterior image of the femur. Head diameter in anteroposterior (HD_AP_) and superoinferior (HD_SI_) planes (Fig 2E) as well as head offset (H_O_) and position (H_P_) (Fig 2D) were measured via ImageJ. H_O_ was defined as the horizontal distance between points at which a line passes through the diaphyseal axis and head center (H_C_). H_P_ was defined as the vertical distance between points at which a line passes through the H_C_ and horizontal level of the lesser trochanter. A medial view of the proximal femur was photographed in order to measure HD_AP_ and HD_SI_. H_O_ and H_P_ were measured from the posterior image of the femur.

Outer diaphyseal diameter (OD) in anteroposterior (AP) and mediolateral (ML) planes was measured with calipers at 25%, 40%, 50%, 60%, and 75% of L_B_. These locations were measured from the distal endpoint of L_B_, 25% being closest the distal end of the femur (Fig 2D and 2E). To measure medullary diameter (MD) in the AP and ML planes (Fig 2D and 2E), the diaphysis was transected with a hacksaw at each of the five marked locations. Prior to transection, the proximal side of each location was marked on the anterior surface to ensure consistent measurement of the proximal piece of bone for each location. Cortical cross-sectional area (CSA), defined as the difference between circular areas formed by OD and MD [4], was determined for each location using ImageJ. Each transected, measured surface (Fig 2B) was photographed with a ruler adjacent to it for scale and camera held parallel to the bench top. OD and MD were outlined using the polygon tool in ImageJ and CSA calculated by subtracting medullary from outer diaphyseal area. Total cortical thickness (CT), defined as the total distance between outer and inner cortices [4], was determined in AP and ML planes (Fig 2B) by subtracting corresponding MD from OD values. AP and ML dimensions of the lateral and medial condyles (LAP, MAP, LML, MML) and intercondylar height (ICH) and width (ICW) were measured by hand as defined in Fig 2C.

Using Excel (Microsoft Excel for Mac 2011, Version 14.4.4 [140807]), means and standard deviations for each dimension for each specimen were calculated and then used to compute overall specimen means. To compare our cervine values with those of human, *Cervus elaphus*, porcine and ovine species, diaphyseal averages of the measured values at 40%, 50% and 60% of L_B_ (mid-diaphysis) were computed for each parameter in AP and ML planes [4]. Average total ML width of the distal femur (fML), defined as the sum of LML, MML and ICW, was calculated [21].

## Results and Discussion

### Mechanical Testing

**Fig 3:**
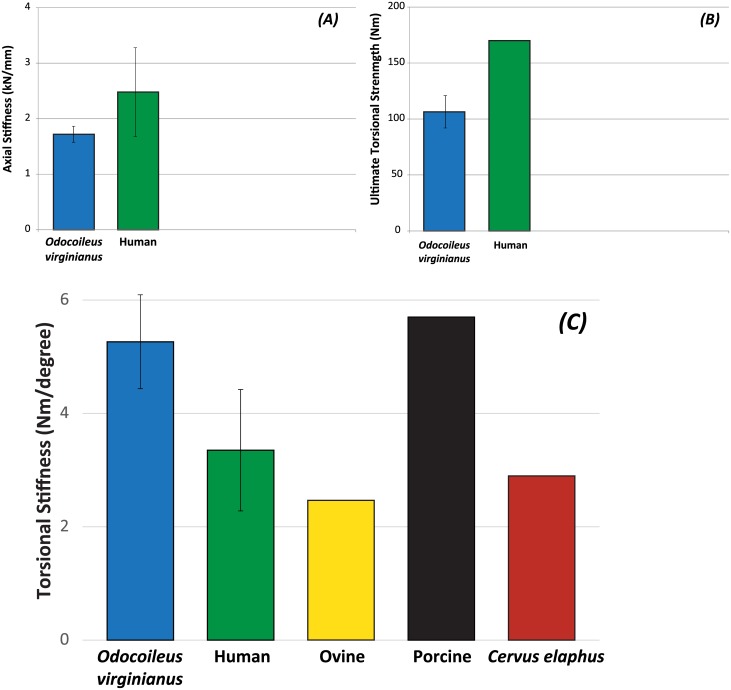
Average axial and torsional stiffnesses (A and C) and ultimate torsional strengths (B) of the *Odocoileus virginianus* (white-tailed deer) femur compared to human [11], ovine (sheep) [4] porcine (pig) [4] and *Cervus elaphus* (red deer) [4] femora. All specimens presented were tested in the same protocol.

All specimens failed under the applied loading. Fig 3 depicts representative examples of fracture patterns and load-displacement curves in both compression and torsion. In comparison to similar measurements from the cervine tibia [14], the load-displacement curve in compression begins more linearly, perhaps due to the shorter overall length of the bone. In addition, the cervine femur fails in torsion at a lower angle of rotation than in the cervine tibia [14]. Fig 3 shows the stiffnesses and torsional ultimate strengths of cervine femora compared to previously reported *Cervus elaphus*, human, ovine and porcine values. Note that the torsional stiffness of the cervine femur is greater than human and ovine femora and comparable to the porcine femur (Fig 3B). Additionally, the cervine femur has an axial stiffness similar to previously reported human and animal values (Fig 3A). Average axial ultimate strength of the *Odocoileus virginianus* femur is 110.3 ± 14.7 Nm. Torsional ultimate strength is within range of human values (Fig 3C).

### Morphology

**Fig 4:**
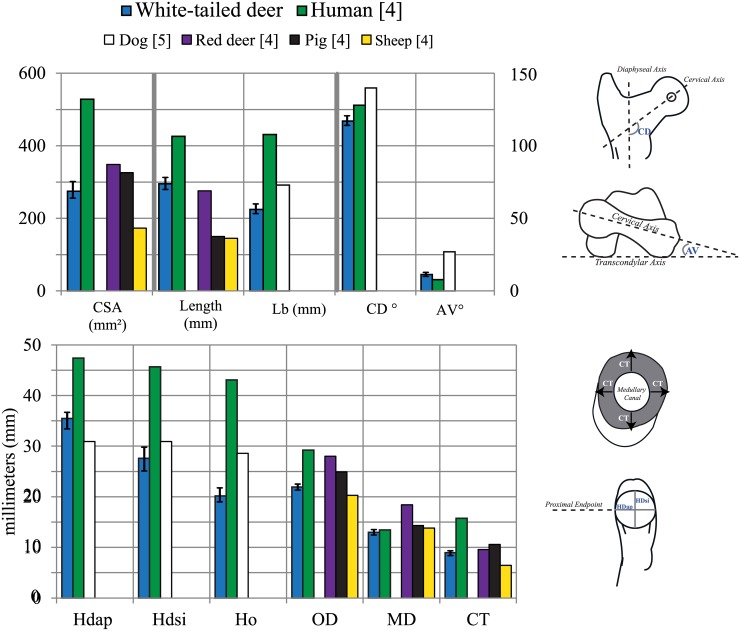
Diaphyseal, head and angular dimension comparison between Odocoileus virginianus (white-tailed deer), human [5, 10], canine [5], *C*. *elaphus* (red deer) [4], porcine [4], and ovine [4] femora. Error bars indicate standard deviation.

**Fig 5:**
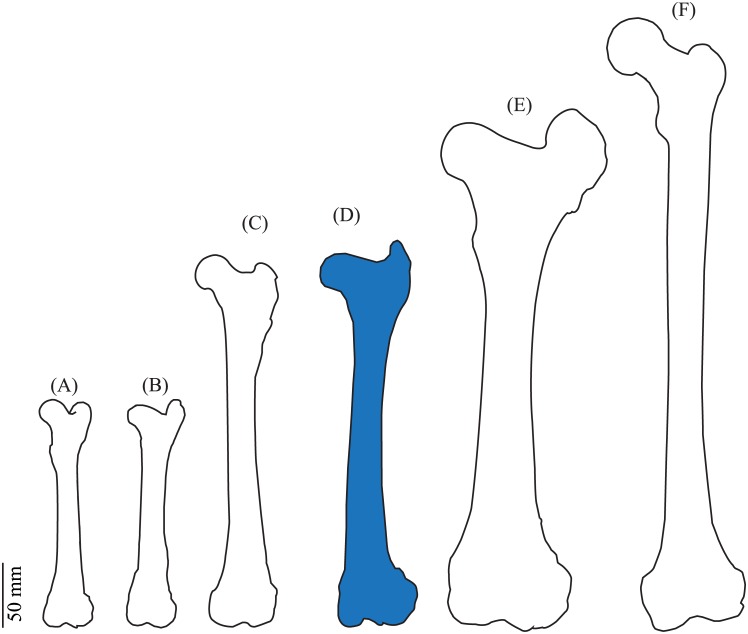
Qualitative comparison of (A) caprine, (B) ovine, (C) canine, (D) cervine, (E) bovine and (F) human femora. Images traced from [23] and scaled to match experimental data.

The average L_T_ and L_B_ of the cervine femur are 295.2 ± 14.0 and 224.8 ± 11.0 mm respectively. When compared with the femur of porcine, ovine and *Cervus elaphus* species, *Odocoileus virginianus* has a femoral length closer to that of humans [4, 10] (Fig 4), which range from 426.0 to 443.0 mm [10]. Fig 5 shows scaled geometric outlines of femora [21, 22] for general size comparison. Average AV and CD angles are 11.4° and 117.0° respectively. These are comparable to yet smaller than average canine values of AV and CD (Fig 4), which were previously reported as 27.0° and 139.8° respectively [5]. The cervine AV angle is closer to that observed in humans (Fig 4). Human values for these angles are 7.8° ± 6.0 for AV and 128.0° ± 8.1 for CD [5]. Femoral head dimensions HD_AP_, HD_SI_, H_O_, and H_P_ of the cervine femur show similar trends to values of the same dimensions of the human femur (Fig 4). Average values for these measurements correspondingly are 35.5 ± 2.0, 27.7 ± 2.4, 20.2 ± 1.8 and 26.2 ± 3.3 mm for *Odocoileus virginianus*, and 47.4 ± 0.7, 45.7 ± 0.7, 43.1 ± 1.8 [5] and 56.0 ± 6.5 mm [20] for humans.

Average cumulative OD of the cervine femur are 23.9 and 22.8 mm in AP and ML planes respectively. These values are comparable to average OD for ovine, porcine, and human femora (Fig 4), which measure 21.1, 25.3, and 29.9 mm in the AP plane and 19.4, 25.3, and 28.5 mm in the ML plane, respectively [4]. *Cervus elaphus* femora have OD values closest to the human whereas porcine and *Odocoileus virginianus* species have MD values closest to the human (Fig 4 and Table 1). Porcine femora appear to have diaphyseal CT most similar to that of humans while *Cervus elaphus* femora have CSA most similar to the human femur.

**Table 1:** Comparison of average femoral diaphyseal dimensions between Odocoileus virginianus, human [10], Cervus elaphus [4], porcine [4], and ovine [4] species.

	*Odocoileus virginianus*	Human	*Cervus elaphus*	Porcine	Ovine
**OD ML**	21.5 ± 1.5	28.5	26.9	24.5	19.4
**OD AP**	22.4 ± 1.5	29.9	29.0	25.3	21.1
**MD ML**	12.9 ± 1.7	14.0	17.8	14.2	13.2
**MD AP**	13.0 ± 1.5	12.9	19.1	14.5	14.4
**CT ML**	8.6 ± 1.0	14.5	9.1	10.4	6.2
**CT AP**	9.3 ± 1.3	17.0	10.0	10.8	6.7
**CSA**	274.7 ± 40.2	528.2	348.6	325.9	173.1

All values are in mm with the exception of CSA, which is in mm^2^.

**Fig 6:**
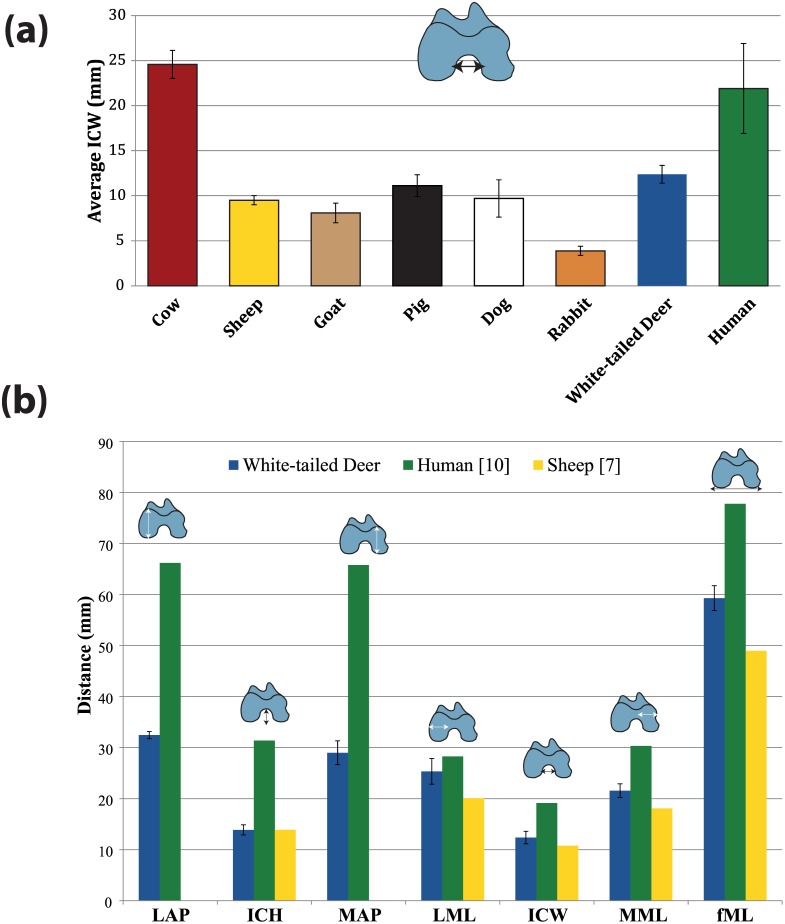
*(a)* Average Intracondylar Width (ICW) of cervine (present study), human [6] and other animal models available in the literature [6] and *(b)* Distal femoral dimension comparison between cervine (Odocoileus virginianus), human [13], and ovine (as available) [12] species. As defined by [21], fML is the total ML width of the distal femur. Error bars represent standard deviation.

Fig 5 depicts distal femoral dimensions of the cervine femur compared to human and ovine species. Average cervine values for LAP, MAP, LML, MML, ICH and ICW are 32.4 ± 2.3, 29.0 ± 2.5, 25.3 ± 1.2, 21.6 ± 1.3, 13.9 ± 0.7 and 12.4 ± 1.0 mm respectively. Previously reported ICH and ICW of ovine species are 13.9 ± 3.4 and 10.8 ± 1.2 mm respectively [12], both of which are comparable to cervine values. When comparing LAP, MAP and fML as defined above, average values for the cervine and human femur are 32.5, 29.0, and 59.3 mm, and 66.2, 65.8 and 77.8 mm [13] respectively. The cervine femur has condylar size closer to that of humans than ovine species (Fig 6).

Biomechanically and morphologically, the *Odocoileus virginianus* femur may be a suitable proxy for the human femur. The combination of the cervine model’s advantageous biomechanical properties and its regional abundance in North America make it a potential tool for further biomechanical research. Cervine femora could advance the design of orthopaedic implants and still provide geometries of interest that other animal models cannot, as well as serve as an inexpensive locally abundant, sustainably-harvested, and inexpensive alternative to human or Sawbone models for ballistics testing, as discussed below.

Both the axial stiffness and ultimate strength of our specimens were lower on average, but within the range of, previously-reported human values. A larger sample size, including only skeletally mature specimens, may bring this average closer to the human values. *Odocoileus virginianus* femora have a higher average torsional stiffness than human femora, but are within one standard deviation of reported human femora torsional stiffness [11]. The *Odocoileus virginianus* femur also has an average torsional stiffness closer to reported human values than both the ovine and porcine femora [4]. While the *Cervus elaphus* femora measured in Kieser *et al*. [11] are even closer to the average human values, we expect that a larger sample of *Odocoileus virginianus* femora would yield similar results. Additionally, the greater torsional stiffness than in human femora is not surprising given the *Odocoileus virginianus* femur’s shorter length.

Morphologically, the cervine femur is markedly shorter than the human femur (average human L_B_ = 431.0 ± 28.3 mm) [5], but closer in length to the human femur compared to other animal models (Figs 4 and 5). *Odocoileus virginianus* femoral length is closer to that of humans than *Cervus elaphus* and some other animals (Figs 4 and 5), and has comparable or more similar diaphyseal dimensions than *Cervus elaphus* (Fig 4, Table 1). Compared to human femur dimensions, the measured cervine dimensions of AV, HD_AP_, HD_SI_, H_O_ and H_P_ follow the same general trends as previously reported canine dimensions (both are smaller or similar to humans for HD_AP_, HD_SI_, H_O_ and H_P_, and larger than humans in AV) [5, 7, 19, 20, 24]. The cervine femoral head has a similar (HD_SI_)/(HD_AP_) ratio to the human femoral head. That is, the cervine femoral head is more ovoid-shaped than the canine femoral head, which is more spherical. This may advance hip arthroplasty research, where canine femora have been a suboptimal primary model [5] due, in part, to the spherical shape of the femoral head. It is interesting to note that the CD angle of cervine femora is smaller than that of humans in contrast to canine, where CD is larger [5, 7, 19, 20, 24]. Both AV and CD angles of the cervine femur are closer to human values than canine species, potentially making cervine femora a better model for hip implants. Previously reported canine AV values ranged from 27.0 to 34.2° and CD from 139.8 to 147.4° [5, 7]. The variation in cervine angular dimensions relative to canine dimensions may offer opportunities in orthopaedic prosthetic device development. The human hip is generally more varus in alignment than that of canine species due to a smaller AV angle[5]. In humans, CD angle is normally between 106 and 140° although it has been measured between 123.0 and 140.0° [19, 20, 24, 25]. Most average CD values for both cervine and canine species fall within this range.

It is noteworthy that cervine femora have ICW dimensions more similar to humans than ovine, caprine, porcine, canine and laprine species (Fig 6a) [6, 12]. The adolescent bovine femur is the only well-studied animal model having an ICW closer to that of humans with an average value of 24.6 mm in one study [6]. In addition, fML of the cervine femur is closer to human dimensions than ovine species (Fig 6b). Therefore, the cervine femur may offer advantageous features over other currently-used quadrupedal animal models for evaluation of knee implants.

The cervine femur has similar biomechanical properties and has average length, angular, distal femoral, diaphyseal and endosteal measurements more similar the human femur than some other animals. Therefore, the cervine femur should be considered for further study as a suitable animal model for the human femur in orthopaedic research and implant design. Its relative size and similar biomechanical properties also make the cervine femur a useful tool for ballistics testing. The results of this study are certainly limited by small sample size, large standard deviations in age estimation, limited scope of the study, and the stochastic nature of the harvesting process. Nonetheless, the cervine femur should be considered for use in biomechanical studies relating to orthopaedic design and ballistic testing. The results of this study may offer an inexpensive, locally abundant and sustainable source of suitable animal models for the human femur in the field of biomechanics.
